# Structure Elucidation and Biochemical Characterization of Environmentally Relevant Novel Extradiol Dioxygenases Discovered by a Functional Metagenomics Approach

**DOI:** 10.1128/mSystems.00316-19

**Published:** 2019-11-26

**Authors:** Chandni Sidhu, Vipul Solanki, Anil Kumar Pinnaka, Krishan Gopal Thakur

**Affiliations:** a Microbial Type Culture Collection and Gene Bank, CSIR-IMTECH, Chandigarh, India; b Structural Biology Laboratory, G. N. Ramachandran Protein Centre, CSIR-IMTECH, Chandigarh, India; Luxembourg Centre for Systems Biomedicine

**Keywords:** halotolerant, oxygenases, fosmid library, aromatic pollutants, X-ray crystallography, dioxygenases

## Abstract

The release of synthetic chemical pollutants in the environment is posing serious health risks. Enzymes, including oxygenases, play a crucial role in xenobiotic degradation. In the present study, we employed a functional metagenomics approach to overcome the limitation of cultivability of microbes under standard laboratory conditions in order to isolate novel dioxygenases capable of degrading recalcitrant pollutants. Fosmid clones possessing dioxygenase activity were further sequenced, and their genes were identified using bioinformatics tools. Two positive fosmid clones, SD3 and RW1, suggested the presence of 2,3-dihydroxybiphenyl 1,2-dioxygenase (BphC-SD3) and catechol 2,3-dioxygenase (C23O-RW1), respectively. Recombinant versions of these enzymes were purified to examine their pollutant-degrading abilities. The crystal structure of BphC-SD3 was determined at 2.6-Å resolution, revealing a two-domain architecture, i.e., N-terminal and C-terminal domains, with the sequential arrangement of βαβββ in each domain, characteristic of Fe-dependent class II type I extradiol dioxygenases. The structure also reveals the presence of conserved amino acids lining the catalytic pocket and Fe^3+^ metal ion in the large funnel-shaped active site in the C-terminal domain. Further studies suggest that Fe^3+^ bound in the BphC-SD3 active site probably imparts aerobic stability. We further demonstrate the potential application of BphC-SD3 in biosensing of catecholic compounds. The halotolerant and oxygen-resistant properties of these enzymes reported in this study make them potential candidates for bioremediation and biosensing applications.

**IMPORTANCE** The disposal and degradation of xenobiotic compounds have been serious issues due to their recalcitrant properties. Microbial oxygenases are the fundamental enzymes involved in biodegradation that oxidize the substrate by transferring oxygen from molecular oxygen. Among oxygenases, catechol dioxygenases are more versatile in biodegradation and are well studied among the bacterial world. The use of catechol dioxygenases in the field is currently not practical due to their aerobically unstable nature. The significance of our research lies in the discovery of aerobically stable and halotolerant catechol dioxygenases that are efficient in degrading the targeted environmental pollutants and, hence, could be used as cost-effective alternatives for the treatment of hypersaline industrial effluents. Moreover, the structural determination of novel catechol dioxygenases would greatly enhance our knowledge of the function of these enzymes and facilitate directed evolution to further enhance or engineer desired properties.

## Abstract (precis)

The disposal and degradation of xenobiotic compounds have been serious issues due to their recalcitrant properties. Microbial oxygenases are the fundamental enzymes involved in biodegradation that oxidize the substrate by transferring oxygen from molecular oxygen. Among oxygenases, catechol dioxygenases are more versatile in biodegradation and are well studied among the bacterial world. The use of catechol dioxygenases in the field is currently not practical due to their aerobically unstable nature. The significance of our research lies in the discovery of aerobically stable and halotolerant catechol dioxygenases that are efficient in degrading the targeted environmental pollutants and, hence, could be used as cost-effective alternatives for the treatment of hypersaline industrial effluents. Moreover, the structural determination of novel catechol dioxygenases would greatly enhance our knowledge of the function of these enzymes and facilitate directed evolution to further enhance or engineer desired properties.

## INTRODUCTION

The industrial revolution is instrumental in providing goods at affordable prices and economic growth, but at the same time hazardous xenobiotic compounds are introduced into the environment as contaminants, causing serious health concerns (1). The disposal and degradation of xenobiotic compounds have been serious issues due to their recalcitrant or nonbiodegradable properties. The existing methods, such as chemical decomposition and high-temperature incineration, are not economical or practical under field conditions (2). Hence, harnessing modern-day bioremediation processes will be suitable and provide potentially harmless alternatives. The process of bioremediation mainly involves the use of microbial or plant enzymes to carry out metabolic transformations of a variety of organic compounds (3). Detoxification of xenobiotic compounds is mediated by intracellular microbial enzymes and is primarily carried out by stimulating microbial growth in contaminated areas (4). Instead of using microbes, even enzymes can be used for bioremediation, but reduced stability and activity under environmental conditions pose a challenge to their effective exploitation. To overcome this limitation and make enzymes more stable for field applications, various strategies, such as enzyme immobilization on solid supports and gel coating, have been developed (5, 6).

Oxygenases play a crucial role in the biodegradation process (7) and are the fundamental enzymes involved in xenobiotic degradation (8). Microbial oxygenases oxidize the substrate by transferring oxygen from molecular oxygen and are important for maintaining the global carbon cycle (9). In addition to biodegradation, oxygenases can also be exploited for industrial processes due to their valuable cleavage products and their tendency to carry out oxygenation of organic molecules in a regio-, stereo-, and chemoselective manner (8). They are broadly classified into monooxygenases and dioxygenases based on the oxygen atoms used during oxygenation (2). Dioxygenases play a central role in biodegradation and are widely distributed among the bacterial population in soil and water (10). Among them, catechol dioxygenases are well studied in the bacterial world (2). They are iron-containing dioxygenases that oxidise catechol or its derivatives (7) and play a central role in the degradation of benzoate and its derivatives (10). Moreover, whole-cell or enzyme-based biosensors could be of great interest in the detection of pollutants in the soil or in industrial waste (11). The use of oxygenases in the field is currently not practical due to their aerobically unstable nature (12). So, the ideal enzyme suitable for field applications should be aerobically stable, halotolerant, and active in broad pH and temperature ranges.

Therefore, in this study, we used a functional metagenomic approach using DNA isolated from sludge and river water samples to isolate and characterize novel catechol dioxygenases capable of efficiently degrading the targeted environmental pollutant. We successfully isolated novel 2,3-dihydroxybiphenyl 1,2-dioxygenase (BphC-SD3) and catechol 2,3-dioxygenase (C23O-RW1). We cloned, expressed, and purified target enzymes and performed biochemical and biophysical characterizations. We also investigated the role of these enzymes in degrading different pollutants and studied the role of metal ions in enzymatic activity. BphC-SD3 was insensitive to the presence of oxygen, while C23O-RW1 did not show any activity in the presence of oxygen under the assay conditions. Since BphC-SD3 is halotolerant and aerobically stable, therefore, we tested its potential as a biosensor capable of detecting catecholic compounds in environmental samples. We were successful in electrochemically detecting the degradation products of these compounds using cyclic voltammetry. We further structurally characterized BphC-SD3 to understand the molecular basis for oxygen insensitivity and to better understand its biological assembly. This study highlights the potential of a functional metagenomic approach in isolating enzymes suitable for targeted applications.

## RESULTS

### Screening of fosmid libraries and description of fosmid clones harboring dioxygenase genes.

Functional metagenomic libraries of sewage sludge and fresh river water consisting of ∼150,000 and ∼70,000 clones, respectively, were screened for the activity of dioxygenases. Five unique fosmid clones tested positive in our screening assays. All of the fosmids were sequenced, and a total of 17 dioxygenase genes were found in both of the libraries. Considering the novelty of the gene sequences, we selected two lower-pathway *meta-*cleavage dioxygenases, namely, BphC-SD3 and C23O-RW1, for recombinant expression and detailed structural and biochemical characterization. These enzymes were present in two dioxygenase-positive clones, SD3 and RW1, prepared from sewage sludge and river water metagenomic libraries, respectively.

SD3 has a G+C content of 63% and a size of 41.5 kb. Out of 44 complete open reading frames (ORFs) found, 37 were successfully assigned to classes according to the Cluster of Orthologous Genes (COG). The detailed description of genes found in fosmid SD3 is given in Table S1 in the supplemental material. The gene annotation suggested that 10 genes had functions related to oxidoreductase activity. The essential genes required for binding of heterocyclic and organic compounds and their transport were detected in fosmid SD3. Further, ion binding and cofactor binding genes required for dioxygenase activity are also present (13). The annotated regulatory genes along with genes involved in aromatic compound catabolism are also present. The genetic organization of fosmid SD3 is shown in Fig. S1A.

10.1128/mSystems.00316-19.1 FIG S1 Genetic organization of fosmids SD3 (A) and RW1 (B) created using DNAPlotter. Download FIG S1, TIF file, 0.1 MB. Copyright © 2019 Sidhu et al. 2019 Sidhu et al. This content is distributed under the terms of the Creative Commons Attribution 4.0 International license.

10.1128/mSystems.00316-19.8 TABLE S1 List of genes present in fosmid clone SD3. Download Table S1, DOCX file, 0.01 MB. Copyright © 2019 Sidhu et al. 2019 Sidhu et al. This content is distributed under the terms of the Creative Commons Attribution 4.0 International license.

The other fosmid, RW1, has a G+C content of 63.4% and a size of ∼34 kb. Out of the 39 ORFs found, 38 were successfully assigned to COG classes. RW1 contains the genes for both upper and lower pathways of aromatic degradation. The genes for the upper pathway include the ring-hydroxylation dioxygenases (RHDs), and the lower pathway contains ring-cleaving dioxygenases. Blast2GO results showed that 7 genes were related to aromatic catabolic process, and 9 genes were assigned to oxidation-reduction functions related to degradation of xenobiotics. A detailed description of genes found in fosmid RW1 is given in Table S2. RHDs present in RW1 showed 94% identity to known RHDs, indicating the conserved nature of upper-pathway enzymes. However, lower-pathway enzymes showed more variability in terms of gene sequence. The genetic organization of fosmid RW1 is depicted in Fig. S1B.

10.1128/mSystems.00316-19.9 TABLE S2 List of genes present in fosmid clone RW1. Download Table S2, DOCX file, 0.01 MB. Copyright © 2019 Sidhu et al. 2019 Sidhu et al. This content is distributed under the terms of the Creative Commons Attribution 4.0 International license.

BphC-SD3 encodes a gene of 891 bp corresponding to a 297-amino-acid protein. A BLAST search using the protein sequence of BphC-SD3 retrieved hits sharing identity of 74% and 72% with glyoxalase of *Sphingomonas* sp. strain MCT13 and *Caulobacteraceae* bacterium OTSz A272, respectively (Fig. S2A). BphC proteins from both species have not yet been characterized. Blast2GO annotations also revealed Pfam, Prosite pattern, and superfamily identifications of BphC-SD3 as InterPro accession numbers IPR004360 (glyoxalase domain), IPR018146 (glyoxalase I, conserved site), and IPR029068 (dihydroxy biphenyl dioxygenase), respectively.

10.1128/mSystems.00316-19.2 FIG S2 Molecular phylogenetic analysis of 2,3-dihydroxybiphenyl 1,2-dioxygenase (BphC-SD3) (A) and catechol 2,3-dioxygenase (C23O-RW1) (B) by the neighbor-joining method. Download FIG S2, TIF file, 0.2 MB. Copyright © 2019 Sidhu et al. 2019 Sidhu et al. This content is distributed under the terms of the Creative Commons Attribution 4.0 International license.

C23O-RW1 is a gene of 912 bp encoding a 304-amino-acid protein. A BLAST search using the protein sequence of C23O-RW1 retrieved hits sharing 91% and 83% identity with 2,3-dihydroxy-*p*-cumate-3,4-dioxygenase and a hypothetical protein of *Polaromonas* sp. and the *Comamonadaceae* family of *Betaproteobacteria*, respectively (Fig. S2B). Blast2GO annotations also revealed the identical Pfam, Prosite pattern, and superfamily identifications of C23O-RW1 as InterPro accession number IPR004360 (glyoxalase domain), Pfam accession number PF00903 (glyoxalase/bleomycin resistance), and InterPro accession number SSF54593 (extradiol dioxygenase), respectively.

### Overexpression and purification of BphC-SD3 and C23O-RW1.

**FIG 1:**
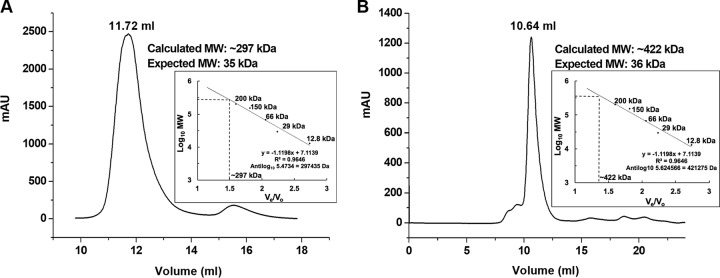
Gel filtration profile of the recombinant BphC-SD3 (A) and C23O-RW1 (B) proteins using an AKTA purifier with a Superdex S-200 10/300 GL increase column. AU, arbitrary units; MW, molecular weight; *V_e_*, retention volume of the protein; *V_o_*, void volume of the column.

BphC-SD3 and C23O-RW1 were cloned in the pET28a vector to yield recombinant proteins having N-terminal 6×His tags. The proteins expressed well in soluble form in Escherichia coli BL21(DE3) cells. Ni-nitrilotriacetic acid (Ni-NTA)-based affinity purification profiles are shown in Fig. 1A and B. As expected, the protein bands for BphC-SD3 and C23O-RW1 on SDS-PAGE were observed at about 35 kDa (Fig. S3). Two-step purification methods using Ni-NTA affinity chromatography followed by gel filtration chromatography resulted in protein samples with >95% purity, as judged by visual inspection of the SDS-PAGE gels. Analytical gel filtration chromatography suggests that BphC-SD3 predominantly exists as an octameric species in solution with an observed molecular weight of ∼300 kDa (Fig. 1A), while the observed molecular weight of ∼420 kDa suggested that C23O-RW1 probably exists as a dodecamer in solution (Fig. 1B). A minor monomer population was also observed in both of the protein samples. Both of the fractions, corresponding to the monomeric and octameric forms, were checked for catechol degradation. A catechol degradation product (2-hydroxymuconic semialdehyde) at 375 nm was observed only in the octameric and dodecameric species of proteins, suggesting that protein oligomerization is essential for activity. Postpurification, C23O-RW1 lost its enzyme activity, with no significant protein degradation observed upon storage at 4°C, suggesting aerobic instability. Hence, further enzyme characterization was carried out using crude samples, as reported earlier (14, 15). Interestingly, BphC-SD3 retained its activity even for months when stored at 4°C. This was an exciting observation as most of the extradiol dioxygenases (EDOs) reported earlier are highly susceptible to oxygen inactivation (16–21). The active fractions of BphC-SD3 were pooled and concentrated up to 3 mg/ml and used for further characterization.

10.1128/mSystems.00316-19.3 FIG S3 SDS-PAGE analysis of BphC-SD3, and C23O-RW1. (A) Purification profile of BphC-SD3. The gel has the following loading order: lane 1, pellet; lane 2, flowthrough; lanes 3 and 4, wash I and II; lane 5, protein ladder; lanes 6 to 9, 20 mM, 100 mM, 200 mM, and 500 mM imidazole elution fractions. (B) Purification profile of C23O-RW1. The gel has the following loading order: lane 1, pellet; lane 2, protein ladder; lane 3, flowthrough; lanes 4 and 5, wash I and II; lanes 6 to 9, 20 mM, 100 mM, 200 mM, and 500 mM imidazole elution fractions. These samples were analyzed by 15% SDS-PAGE. Download FIG S3, TIF file, 0.3 MB. Copyright © 2019 Sidhu et al. 2019 Sidhu et al. This content is distributed under the terms of the Creative Commons Attribution 4.0 International license.

### Substrate specificity of BphC-SD3 and C23O-RW1.

**FIG 2:**
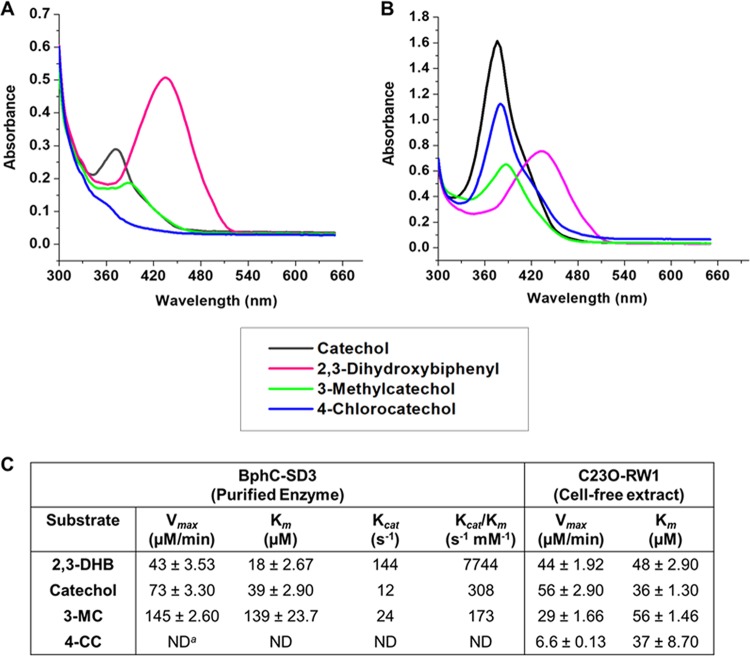
Degradation of different catecholic substrates by BphC-SD3 (A) and C23O-RW1 (B). All enzyme activity measurements were determined by the formation of products at the following wavelengths: 2,3-dihydroxybiphenyl (2,3-DHB), 434 nm; catechol, 375 nm; 3-methylcatechol (3-MC), 388 nm; 4-chlorocatechol (4-CC), 379 nm. (C) Kinetic constants of BphC-SD3 and C23O-RW1. Values are means ± standard deviations. ND, not determined.

The relative activities of BphC-SD3 and C23O-RW1 toward different catecholic (2,3-dihydroxybiphenyl [2,3-DHB], catechol, 3-methylcatechol [3-MC], 4-chlorocatechol [4-CC], 1,2-dihydroxynaphthalene, 1,3-dihydroxynaphthalene, 4-nitrocatechol, and 3,5-dichlorocatechol) and other substrates (pyrogallol and gentisic acid) were examined. BphC-SD3 was found to be efficient and specific in oxidizing 2,3-DHB, catechol, and 3-MC but was unable to degrade 4-CC and other compounds mentioned above (Fig. 2A). Enzyme kinetics data suggest that among the examined substrates, BphC-SD3 preferably cleaves 2,3-DHB. The catalytic efficiency (*k*_cat_/*K_m_*) of BphC-SD3 with 2,3-DHB was found to be ∼25-fold higher than that of other substrates, suggesting its preference for bicyclic substrates (Fig. 2C). Data suggest that BphC-SD3 cleaves substrates in the order 2,3-DHB > catechol > 3-MC. The *k*_cat_/*K_m_* of BphC-SD3 for 3-MC was more than 3 orders of magnitude lower than that for 2,3-DHB, showing its low catalytic efficiency in cleaving 3-MC.

C23O-RW1 was found to be efficient and specific in oxidizing catechol, followed by 4-CC, 2,3-DHB, and 3-MC, but was unable to oxidize the noncatecholic compounds (Fig. 2B). As C23O-RW1 is sensitive to oxygen, the maximum rate of metabolism (*V*_max_) and *K_m_* were determined using cell-free extracts of E. coli cells overexpressing C23O-RW1, as described in previous studies (14, 22). The substrate specificity of C23O-RW1 is in the order catechol > 4-CC > 2,3-DHB > 3-MC (Fig. 2C).

### Effect of temperature and pH on enzyme activity.

**FIG 3:**
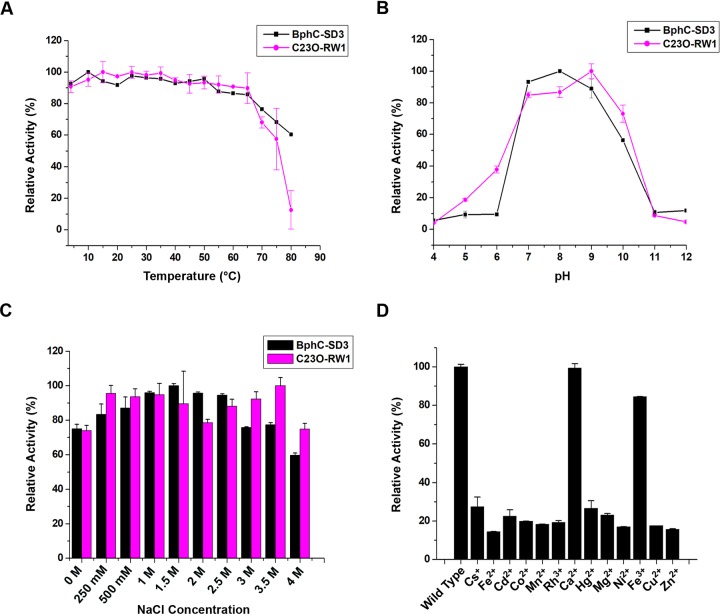
(A) Graph depicting the stability of BphC-SD3 and C23O-RW1 at different temperatures (4°C to 80°C). Relative activity was measured after incubating the enzymes at different temperatures for 30 min. (B) Relative activity of BphC-SD3 and C23O-RW1 at pH values ranging from pH 4 to pH 12. (C) Biocatalytic activity of BphC-SD3 and C23O-RW1 in the presence of different concentrations of NaCl. (D) Percent reactivation of apo-BphC-SD3 in the presence of various metal ions at a 1 mM concentration.

The effects of temperature on the enzyme activity of both proteins were examined at a temperature range of 0 to 80°C using 2,3-DHB as the substrate. Results showed that both enzymes have moderate thermal stability and retained ∼85% activity when preincubated at 65°C for 30 min. BphC-SD3 showed broad-range thermal stability and retained ∼60% relative activity even at 80°C whereas negligible activity was observed when C23O-RW1 was preincubated at 80°C for 30 min (Fig. 3A).

The effect of pH on enzyme activity was measured using different buffers with pH values ranging from pH 4 to pH 12. BphC-SD3 showed maximum activity at pH 8, with an optimum pH range of pH 7 to pH 9. About 90% relative activity was observed at pH 7 and pH 8. C23O-RW1 showed maximum activity at pH 9; however, ∼80% activity was retained at pH 7 and pH 8 (Fig. 3B). No activity was observed under extreme acidic and basic conditions in either enzyme.

### Effect of salinity on BphC-SD3 and C23O-RW1.

To determine the effect of salinity on BphC-SD3 and C23O-RW1, NaCl concentrations ranging from 0 M (control) to 4 M were used. The data suggest that both enzymes showed improved activity with increasing salt concentration (Fig. 3C). BphC-SD3 showed maximal activity at 1.5 M NaCl (*P = *0.0001) whereas C23O-RW1 was found to retain maximal activity at 3.5 M NaCl (*P = *0.0015), suggesting the halophilic nature of both enzymes. Incubation of enzymes for 12 h at 25°C in the presence of 1.5 M NaCl resulted in only a minimal loss in enzyme activity.

### Effect of metal ions on BphC-SD3 and C23O-RW1.

The effect of metal ions was determined by incubating the apoenzyme with different metal ions. Percent reactivation data suggest that BphC-SD3 is highly active in the presence of Ca^2+^ ions, showing ∼99% reactivation relative to levels with the holoenzyme whereas ∼83% relative activity was regained in the presence of Fe^3+^ ions. However, only 15% to 25% of relative activities were obtained in the presence of other metal ions tested in this study (Fig. 3D). Data presented here suggest that BphC-SD3 can effectively use either Ca^2+^ or Fe^3+^ to perform catalysis. However, activity was not restored when apo-C23O-RW1 was incubated in the presence of different metals ions.

### Structure determination and the oligomeric state of BphC-SD3.

BphC-SD3 crystallized under several conditions. Crystals of BphC-SD3 appeared in 2 to 3 days under several conditions of a ProPlex screen (Molecular Dimensions, Ltd., United Kingdom). The diffraction-quality crystals were grown in 0.1 M sodium citrate, pH 6.0, and 2 M NaCl. The crystals diffracted to about 2.6-Å resolution, and the structure was solved by a molecular replacement method. The data collection, processing, and refinement statistics are given in Table 1.

**TABLE 1:** Data collection and refinement statistics obtained for BphC-SD3

Parameter	Value for BphC-SD3^*a*^
Data collection	
Space group	I422
Cell dimensions	
*a, b, c* (Å)	98.64, 98.64, 158.56
*α, β, γ* (°)	90.00, 90.00, 90.00
Resolution (Å)	31.19–2.60
* R*_merge_	0.141 (0.509)
* I*/σ*I*	10.1 (3.3)
Completeness (%)	99.8 (99.7)
Redundancy	5.3 (5.3)
Wavelength (Å)	1.5418
Refinement statistics	
Resolution (Å)	31.19–2.60
No. of unique reflections	12,415
*R*_free_ test set (%)	5
*R*_work_/*R*_free_	0.194/0.248
No. of atoms	
Protein	2,344
Water	151
Ligand	1
B-factor (Å^2^)	
Protein	25.21
Water	18.88
Ligand	56.74
RMSD^*b*^	
Bond length (Å)	0.008
Bond angle (°)	1.18
Structure validation	
Ramachandran plot statistics (%)	
Most favored	96.62
Allowed region	3.38
Disallowed region	0

a Values in the parentheses correspond to the highest-resolution shells.

b RMSD, root mean square deviation.

**FIG 4:**
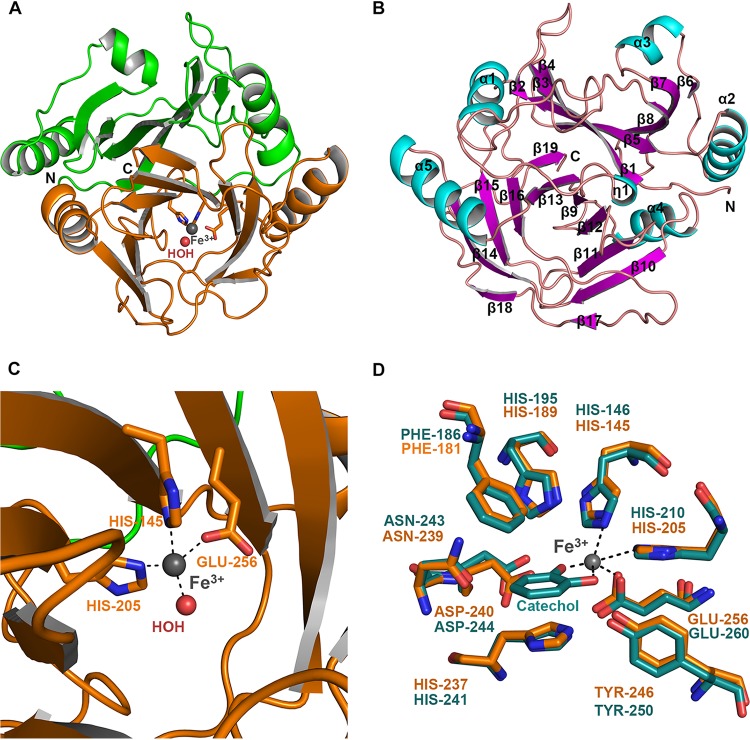
Crystal structure of BphC-SD3. (A) Cartoon representation of BphC-SD3 displaying N- and C-terminal domains in green and orange, respectively. (B) Cartoon representation of BphC-SD3 displaying secondary structure elements and N and C termini. (C) Highly conserved residues His145, His205, Glu256, and a water molecule coordinate Fe^3+^ metal ion, forming a tetradentate geometry. (D) Structural superposition of conserved active-site residues, shown in stick representation, of catechol 2,3-dioxygenase solved in the presence of catechol (PDB ID 1KND; green) and BphC-SD3 (orange).

The crystal belonged to the I422 space group, and there was one molecule in the asymmetric unit. All of the residues from 1 to 296 could be modeled in the electron density. The monomer is composed of topologically similar domains, i.e., an N-terminal domain (residues Met1 to Thr138) and a C-terminal domain (Gly139 to Asn296), including a 12-residue-long C-terminal tail (Fig. 4A). The overall structural fold of BphC-SD3 was similar to structural folds of members of the class of extradiol dioxygenases (23). The structure consists of 6 α-helices and 19 β-strands which are arranged in the order β1-α1-β2-β3-β4-β5-α2-β6-α3-β7-β8-η1-β9-α4-β10-β11-β12-β13-α5-β14-β15-β16-β17-β18-β19 (Fig. 4B). The N-terminal domain is composed of 8 β-strands and 3 α-helices, while the C-terminal catalytic domain is composed of 11 β-strands and 3 α-helices. Apart from two classical βαβββ motifs in each N- and C-terminal domain, BphC-SD3 has three extra β-strands (β17 to β19) in the extended C-terminal tail. Additionally, it also has one extra α3 helix in the N-terminal domain, which is not part of the typical βαβββ motif.

The enzyme BphC-SD3 is a dimer of tetramers. Two layers of tetragonally arranged subunits are rotated ∼45° relative to each other. This kind of similar arrangement was observed in 2,3-dihydroxybiphenyl 1,2-dioxygenase from *Pseudomonas* sp. strain LB400 (17, 24). The octamer is a compact globular molecule having a central pore with maximum and minimum diameters of 43 Å and 19 Å, respectively. It has 422 (D4) symmetry and a maximum dimension of ∼90 Å. There is a 12-residue extension present at the C terminus of BphC-SD3 which adopts the β-sheet conformation. This C-terminal β-strand is present at the opening of the active site and is not involved in any intersubunit interactions. Four residues of these 12 residues form the β19 strand arranged antiparallel to the β13 strand. Overall, BphC-SD3 structure is organized in two domains, an N-terminal domain and a C-terminal domain. Each domain includes mixed β-sheets partially covering a large funnel-shaped space present entirely within the C-terminal domain. The active-site metal ion binds within this space. The domain-domain interfaces are formed by corresponding strands from β-sheets of the two domains as well as surface helices. Interestingly, sequential arrangement of βαβββ in each domain gives rise to four such copies in the whole structure, suggesting probable gene duplication at two points in the evolutionary history of the enzyme. Similar structural features have been reported for the BphC from Pseudomonas cepacia (25), BphC from *Pseudomonas* sp. strain KKS102 (26), the metapyrocatechase from Pseudomonas putida mt-2 (27), and homoprotocatechuate 2,3-dioxygenase (HPCD)-containing Fe^2+^ and Mn^2+^ ions (28). This domain represents the vicinal oxygen chelate (VOC) superfamily of metalloproteins and is indicative of type I EDO. The two-domain arrangement shows that BphC-SD3 is a class II type I EDO. The examination of the protein sequences of EDOs with monomers of relative molecular weights of ∼35 kDa suggests that a two-domain architecture is a typical feature of many Fe-dependent enzymes that carry out *meta*-cleavage of aromatic rings. So far, the analysis suggests that metal binding and catalytic activity are not likely to be present in the N-terminal domains of the enzymes characterized to date.

### The catalytic site of BphC-SD3.

The multiple sequence alignment of structurally characterized dioxygenases having <60% sequence identity with BphC-SD3 showed that the residues interacting with iron are conserved. (Fig. S4). In Fig. 4C the iron is shown to have tetrahedral coordination involving His-145, His-205, and Glu-256 protein ligands and one water molecule. The detailed geometry of the coordination sphere is shown in Table S3a. Structural superimposition of BphC-SD3 with the structure of Protein Data Bank identification number (PDB ID) 1KND (24), which has catechol present in the active site, shows that while the conserved residues interacting with iron are still conserved, the catechol has replaced the two water molecules, and two hydroxyl groups are forming bonds with iron (Fig. 4D). To identify the catalytic site of BphC-SD3, superimposition of BphC-SD3 was performed with other known dioxygenases such as Burkholderia xenovorans (PDB ID 1HAN, root mean square deviation [RMSD] of 0.8 Å for 253 C^α^ atoms; PDB ID 1KND, RMSD of 0.8 Å for 254 C^α^ atoms), *Pseudomonas* sp. strain KKS102 (PDB ID 1EIL, RMSD of 0.88 Å for 249 C^α^ atoms), and *Pseudomonas* sp. strain C18 (PDB ID 2EHZ, RMSD of 1.5 Å for 245 C^α^ atoms) that were bound with the substrate (24–26). This analysis revealed that the residues forming the catalytic site were well conserved in BphC-SD3, suggesting that it may display a similar catalytic mechanism. The closest structural homologs of BphC-SD3 were obtained using the PDBeFOLD server (http://www.ebi.ac.uk/msd-srv/ssm/cgi-bin/ssmserver). The top hit was 2,3-dihydroxybiphenyl 1,2-dioxygenase (DHBD) of B. xenovorans (PDB ID 1HAN, having 40% sequence identity) with an RMSD of 0.8 Å over 253 C^α^ atoms. The analysis demonstrated that the conserved domains, including Fe ligands (His145, His205, and Glu256), and eight residues forming the inner channel wall of the active-site cavity were all consistent in the aligned sequences (Fig. S5). A closer view in Fig. S5 illustrates the essential features of the active site in substrate-free states of the enzyme. The catalytic pocket is formed by His-145, Phe-181, His-189, His-205, His-237, Asn-239, Asp240, Tyr-246, and Glu-256. In particular, the evolutionarily conserved active site of the novel BphC-SD3 was identical to classic active sites, all of which had been previously structurally studied (25), validating that the novel enzymes could be assigned to dioxygenase. The strains reported above, *Pseudomonas* sp. strain LB400, P. cepacia, and Burkholderia xenovorans, are identical, and the current acceptable taxonomic nomenclature is B. xenovorans.

10.1128/mSystems.00316-19.4 FIG S4 Multiple sequence alignment of amino acid sequence of C-terminal domains of BphCs sharing <40% sequence identity. Metal-interacting conserved residues His145, His205, and Glu256 are indicated by black arrow. Download FIG S4, TIF file, 0.9 MB. Copyright © 2019 Sidhu et al. 2019 Sidhu et al. This content is distributed under the terms of the Creative Commons Attribution 4.0 International license.

10.1128/mSystems.00316-19.5 FIG S5 Structural superposition of conserved active-site residues, shown in stick representation, of close homologs (PDB 1HAN, cyan; 1KND, forest green; 1EIL, green; 2EHZ, magenta) with BphC-SD3 (orange). The residues interacting with the metal ion F^e3+^ are shown with their distance from Fe^3+^. Water, red sphere; Fe^3+^, grey sphere. Download FIG S5, TIF file, 0.4 MB. Copyright © 2019 Sidhu et al. 2019 Sidhu et al. This content is distributed under the terms of the Creative Commons Attribution 4.0 International license.

10.1128/mSystems.00316-19.10 TABLE S3 (a) Detailed geometry of coordination sphere of BphC-SD3. (b) Active-site metal ions and coordinating residues in EDOs. Download Table S3, DOCX file, 0.02 MB. Copyright © 2019 Sidhu et al. 2019 Sidhu et al. This content is distributed under the terms of the Creative Commons Attribution 4.0 International license.

### BphC-SD3 forms an octameric assembly in solution.

**FIG 5:**
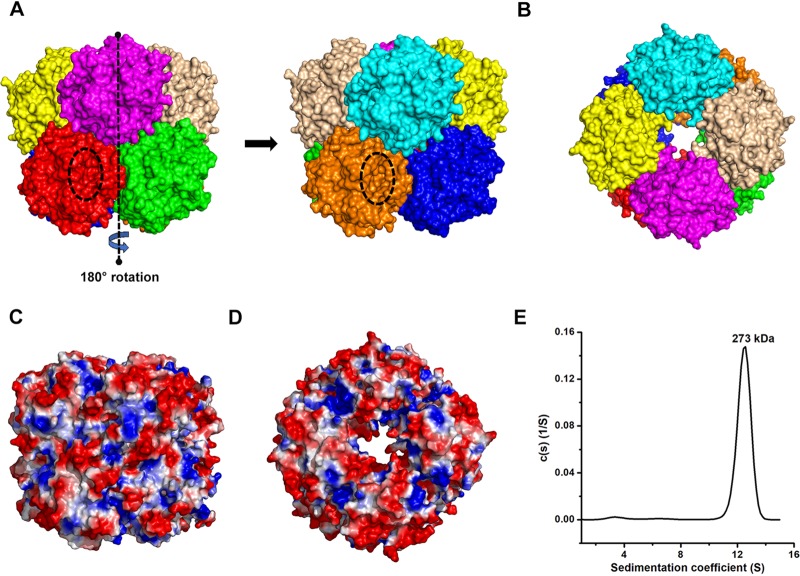
Octamer of BphC-SD3 generated using crystal symmetry. (A) Side views. (B) Top view showing a central pore having maximum and minimum diameters of 43 Å and 19 Å, respectively, created as a consequence of self-association. The assembly shows BphC-SD3 as the homodimer of two tetramers. Surface representations of BphC-SD3 as a (C) monomer and (D) octamer are shown. The positive charges are shown in blue, negative charges are shown in red, and neutral charges are shown in white. (E) AUC analysis of BphC-SD3. A single peak in AUC suggested a monodisperse sample. The observed molecular weight of ∼273 kDa corresponds to the octameric oligomeric state of BphC-SD3.

Size exclusion chromatography (SEC) suggested an octameric oligomeric state of BphC-SD3 in solution. Crystal structure analysis revealed that an octameric assembly formed a dimer of tetramers where ring-shaped tetramers are stacked on each other (Fig. 5A). The PDBePISA analysis of BphC-SD3 showed that the total surface area of octamer is about 83,000 Å^2^, which comprises a buried surface area of about 17,500 Å^2^. The subunits in each ring are stabilized by hydrogen bonds and salt bridge interactions while two rings are maintained by hydrophobic and ionic interactions. The Arg241-Glu45, Thr272-Gly119, Thr272-Gln120, Glu162-Lys71, Glu269-Arg3, Glu269-Thr68, Gly280-Asp221, His214-Cys236, Lys218-Asn125, Pro132-Gln128, Gln128-Pro129, Gln128-Trp131, and Thr272-Val122 residues participate in hydrogen bond formation, and residues Arg241-Glu45, Glu162-Lys71, and Glu269-Arg3 form salt bridges. The hydrogen bonds and salt bridges were observed playing a critical role in forming the surface interactions that keep the octamer functionally active. The octamer assembly leads to the formation of a large pore in the center with wide openings having minimum and maximum diameters of 19 Å and 43 Å, respectively. The dimensions of the octamer assembly are a width of about 90 Å width and length of about 98 Å (Fig. 5B). However, all eight catalytic sites are present on the surface of the octamer very distant from the central pore. The electrostatic potential maps of BphC-SD3 are displayed in Fig. 5C and D and reveal a highly acidic surface.

We further confirmed the oligomeric state of BphC-SD3 by performing analytical ultracentrifugation (AUC) experiments. The acquired data were analyzed using SEDFIT (29). The resulting continuous size distribution profile, *c*(*s*), is shown in Fig. 5D, and the data were fit with an RMSD of 0.009158. The AUC data analysis suggests the presence of a single predominant population of protein with a sedimentation coefficient (*s*) of 12.4, corresponding to a molecular weight of ∼273 kDa. This molecular mass corresponds very well with the theoretically calculated mass of the BphC-SD3 octamer based on the amino acid sequence of each subunit, suggesting the formation of an octameric assembly. The weight-average frictional ratio (*f*/*o*) was optimized by least-squares regression and converted to a best-fit value of 1.3, indicating globular conformation of the BphC-SD3 octamer.

### Sequence analysis and molecular modeling of C23O-RW1.

Catechol 2,3-dioxygenase (C23O-RW1) was categorized as an extradiol dioxygenase, and its structure was modeled using the I-TASSER server (30–32). The modeled structures showed a typical dioxygenase domain arrangement, with an N-terminal domain and C-terminal domain (Fig. S6A). The sequence identity between C23O-RW1 and BphC-SD3 was 25%. The protein C23O-RW1 has an additional 5 residues as the C-terminal tail compared to the sequence of BphC-SD3. Sequence alignment with BphC-SD3 showed that conserved residues interacting with iron, namely, His-145, His-205, and Glu-256, were conserved in both proteins (Fig. S6C). The surface of the protein was found to be highly acidic in nature (Fig. S6B).

10.1128/mSystems.00316-19.6 FIG S6 (A) Structural superposition of C23O-RW1 (cyan) with BphC-SD3 (orange). (B) Electrostatic charge surface representation of C23O-RW1. The negative charges are shown in red, positive charges are shown in blue, and neutral charges are shown in white. (C) Multiple sequence alignment of amino acid sequences of C23O-RW1 and BphC-SD3. Metal-interacting conserved residues His145, His205, and Glu256 are highlighted in violet. Only the region of interest is shown in sequence alignment. Download FIG S6, TIF file, 0.5 MB. Copyright © 2019 Sidhu et al. 2019 Sidhu et al. This content is distributed under the terms of the Creative Commons Attribution 4.0 International license.

### Exploiting BphC-SD3 for biosensing of catecholic compounds.

**FIG 6:**
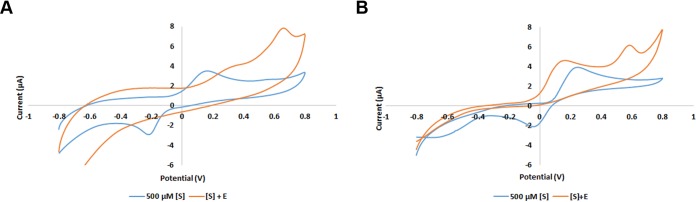
Cyclic voltammetry profile of catechol (A) and 3-methylcatechol (B) at a scan rate of 100 mV/s. [S], substrate concentration; E, enzyme concentration. The profiles explain the detection of catechol and 3-methylcatechol by recombinant enzymes using screen-printed electrodes.

Considering aerobic and operational stability, we wanted to test the potential of BphC-SD3 in biosensing of catecholic compounds. To confirm the constant voltage and current in all the experiments, a scan rate of 0.1 to 1 mV/s was applied, and the results showed uniformity across all the scan rates (Fig. S7). The reversible redox peaks were observed in the case of substrates in the absence of an enzyme. This is possibly due to the reversible quinine redox of catechol and 3-MC. However, the electrochemical behaviors of catechol and 3-MC are quite different in the presence of BphC-SD3 as no reduction peak was observed. The reduction peaks disappeared with the positive shifts in the oxidation peaks. In the case of catechol, a significant voltage difference (Δ*V* of ∼200 mV) was observed, and in the case of 3-MC, two oxidation peaks, probably due to the oxidation of two functional groups, were observed (Fig. 6). Electrochemical data along with the kinetics analyses suggest that BphC-SD3 is capable of performing efficient oxidation of catecholic substrates and, hence, could be exploited in developing enzyme-based biosensors for the sensitive detection of catecholic compounds. However, more experiments need to be conducted for the synthesis of a reliable and portable biosensor.

10.1128/mSystems.00316-19.7 FIG S7 Standard graphs showing the constant voltage and current during cyclic voltammetry experiments of degradation of catechol (A) and 3-MC (B). Download FIG S7, TIF file, 0.1 MB. Copyright © 2019 Sidhu et al. 2019 Sidhu et al. This content is distributed under the terms of the Creative Commons Attribution 4.0 International license.

## DISCUSSION

Bioremediation of industrial pollutants through detoxification and mineralization by microbial pathways has received tremendous attention from researchers. Thus, these microbial pathways that degrade aromatic compounds are being targeted for discovering and developing enzyme-based bioremediation processes. In aromatic compound degradation by aerobic pathways, the activation of a benzene ring by hydroxylation of adjacent carbons is the first step to form intermediates such as catechol (33). The intermediates are then oxidized by specific enzymes called dioxygenases. These aromatic ring cleavage dioxygenases belong to three large families, i.e., (i) iron (Fe^2+^/Fe^3+^)-containing, (ii) cofactor-independent, and (ii) cambialistic dioxygenases that contain other divalent metal ions, e.g., copper, nickel, manganese, or cobalt, in their reaction centers (34–37). Based on the position of cleavage, dioxygenases are further classified into intradiol dioxygenases, with cleavage between the hydroxylated carbon atoms (*ortho*-cleavage), and extradiol dioxygenases, with cleavage at the carbon-carbon bond adjacent to hydroxyl groups (*meta*-cleavage) (33). EDOs utilize Fe(II) as a cofactor in a mononuclear nonheme, nonsulfur environment and are pioneering subjects for studying the pathway evolution and enzyme development (38, 39). The extradiol dioxygenases are classified in three classes, I to III, of extradiol-cleaving catecholic dioxygenases based on the amino acid sequence (40). A comparison of primary structures of these enzymes has revealed that the class II enzymes have evolved through gene duplication from a class I enzyme (40, 41) while class III enzymes have no sequence similarity to the class I and II enzymes. To date, crystal structures of class II and class III have been determined (25, 27, 42–44). We determined the crystal structure of BphC-SD3 (class II) and observed that amino acid residues around the catalytic pocket are conserved among the class II enzymes. The enzyme LigAB, protocatechuate 4,5-dioxygenase, is derived from Sphingomonas paucimobilis SYK-6 and is classified as a class III extradiol dioxygenase. Moreover, the active-site structures of BphC-SD3 and LigAB are very similar even though there is no overall structural similarity. This similarity suggests a convergent evolution between class II and III enzymes and that they may share similar catalytic mechanisms (44).

In recent decades, the metagenomic approach has emerged as a powerful strategy to discover enzymes with outstanding industrial applications or ecological solutions. Few studies have been performed so far which were based on the preparation of a metagenomics library from polluted soil samples or sludge samples (45–47). In the present study, using a functional metagenomics approach, we successfully discovered two EDOs, one each from sewage sludge and fresh river water, namely, BphC-SD3 and C23O-RW1, respectively, and performed detailed biochemical and biophysical characterizations. Our data suggest that BphC-SD3 is insensitive to the presence of oxygen and could perform efficient catalysis even when stored for months at 4°C. So, to understand the molecular basis for this, we determined the crystal structure of BphC-SD3.

Based on the crystal structure, structural superimpositions, and sequence alignment analysis, BphC-SD3 and C23O-RW1 were categorized as EDOs. The EDOs are known to utilize both monocyclic substrates and bicyclic substrates (48). BphC-SD3 has a relaxed substrate-degrading range and is capable of degrading 2,3-DHB, 3-MC, and catechol. This may be due to the larger substrate binding pocket cavity observed in the crystal structure. As reported in earlier studies, dioxygenases are inactivated by chlorocatechols (49), but, surprisingly, C23O-RW1 is capable of cleaving 4-CC efficiently.

The extradiol-type dioxygenases typically contain one nonheme iron [Fe(II)] in their active sites but were also found to be active with Mn(II) (28, 50). The iron Fe(III) center of the substrate-free form of aerobically purified BphC derived from *Pseudomonas* sp. strain KKS102 can be described as a five-coordinated square pyramidal center with one axial (His145) and four basal (His209, Glu260, and two water molecules) ligands (25, 43, 51, 52). The structure of the same inactive enzyme BphC with substrate showed a trigonal bipyramidal iron center where equatorial ligands are His145, Glu260, and a hydroxy group of the substrate; the axial ligands are His209 and another hydroxyl group of the substrate (43). Unlike BphC, BphC-SD3 is active in the presence of oxygen, and the iron Fe(III) center is tetradentate pyramidal, with a His145 axial ligand and three basal ligands (His205, Glu256, and a water molecule). His194 (His189 in BphC-SD3) is indispensable for the catalytic activity, and the shape of the substrate-binding pocket and its hydrophobic character are important for the substrate binding (53).

Our metal ion studies suggested that BphC-SD3 is capable of performing catalysis in the presence of Ca^2+^ or Fe^3+^. However, a brown tinge was observed while concentrating BphC-SD3, suggesting probably the presence of iron in the active site of the natively purified enzyme. In previous studies, upon oxidation, active Fe(II)-containing EDOs, which are purified anaerobically, turn brown, indicating inactive Fe(III)-containing EDOs (44, 53, 54). To date, no Ca^2+^-containing EDOs have been reported. This is quite interesting as EDOs are characterized by the presence of Fe(II) in their reaction centers, suggesting that Bphc-SD3 is a noncanonical EDO. EDOs lose their activity in the presence of oxygen due to iron oxidation (16, 17, 19, 55). In contrast, BphC-SD3 remains highly active even in the presence of oxygen. The requirement of Ca^2+^ or Fe^3+^ probably explains the oxygen insensitivity of BphC-SD3. However, this needs to be tested if other EDOs can accept Ca^2+^ in the active site and, hence, can remain insensitive to the presence of oxygen. We analyzed the crystal structures of different metal-bound EDOs and observed that three highly conserved residues, namely, His, His, and Asp, are involved in metal coordination (see Table S3b in the supplemental material). Hence, no observable relationship was observed between the metal ion or the metal-coordinating amino acids in the active site. Emerson et al., have shown that, despite possessing two different metal ions, Fe^2+^-bound homoprotocatechuate 2,3-dioxygenases from Brevibacterium fuscum (Fe-HPCD) and Mn^2+^-bound HPCD from Arthrobacter globiformis (Mn-HPCD) have similar active-site structures and nearly the same kinetic parameters (56). Cleavage patterns of EDOs can be altered by mutating active-site residues (57, 58). However, a typical extradiol (*meta*-) cleavage yellow product was observed upon cleavage by BphC-SD3. There is one report according to which an Fe(III)-containing intradiol dioxygenase has been shown to perform extradiol cleavage (59). Our structural analyses as well as multiple sequence alignment analyses revealed the evolutionarily conserved active-site residues (His-145, His-205, and Glu-256) in BphC-SD3, suggesting that BphC-SD3 is an extradiol dioxygenase. Together, all of these results should greatly enhance our knowledge of the function of these enzymes and facilitate the directed evolution to enhance or engineer desired properties.

Wastewater effluents discharged from many industries, e.g., leather and textile industries, tanneries, and drinking water treatment processes, are highly saline, and they are known to adversely affect agriculture as well as aquatic life (60, 61). The treatment of hypersaline industrial effluents includes physiochemical treatments that are highly expensive (62). A halotolerant enzyme-based bioremediation system can provide a cost-effective alternative. Both BphC-SD3 and C23O-RW1 are halotolerant and can tolerate upto 4 M NaCl. The use of a microbial consortia harboring dioxygenases for the treatment of hypersaline industrial effluents has already been described in previous studies (63–65). This property makes our enzymes an attractive alternative to efficiently remediate salt marshes and hypersaline industrial wastewaters.

Electrochemical detection of pollutants using enzymes is becoming increasingly popular due to the ease and speed of detection. Low-cost screen-printed electrodes (SPE) have been reported for detecting pollutant degradation using cyclic voltammetry and differential pulse voltammetry (66). In 2012, Zhang et al. developed a 2,3-dihydroxybiphenyl 1,2-dioxygenase (BphC)-based method for the detection of 2,3-dihydroxybiphenyl and catechol using CdTe-quantum dots (1). The same enzyme (BphC) was used by another group to construct a polyvinyl alcohol-modified SiO_2_ sol gel biosensor on a glassy carbon electrode (67). The aerobic stability of BphC-SD3 alleviates the need to immobilize it and, hence, offers fast and reliable detection of cleavage products of catechol and 3-MC. The current study demonstrates excellent possibilities for monitoring pollutants in real contaminated samples using BphC-SD3. However, the method needs to be tested rigorously to evaluate its suitability under field conditions.

In summary, we were successful in isolating novel halotolerant dioxygenases using a functional metagenomics approach that have immense potential in bioremediation, biosensing, or other chemical synthesis applications. The results presented here can also be utilized to engineer dioxygenases active under aerobic conditions to improve their potential for field or biosensing purposes. The functional metagenomics-based approach, presented here, holds immense potential for fishing out enzymes suitable for biotechnological, industrial, and research applications.

## MATERIALS AND METHODS

### Metagenomic library preparation, screening, and identification of EDOs.

Two functional metagenomic libraries from sewage sludge (30°41′12.335′′N, 76°49′20.497′′E) and fresh river water (31°01′12′′N, 76°30′ 0′′E) were prepared to screen for the extradiol dioxygenase (EDO) function. Briefly, high-molecular-weight DNA (∼40 kb) was isolated from the mentioned samples using a Meta-G-nome DNA isolation kit (Epicentre), and the functional metagenomic library was prepared in the pCC2FOS vector (Epicentre) according to the manufacturer’s instructions. Briefly, E. coli EPI300 phage-resistant cells (Epicentre) were used for transformation, and CopyControl induction solution provided with the cells was used to induce the genes of interest. The transformed cells were incubated at 37°C on an LB plate with 12.5 μg/ml chloramphenicol for 36 h. After incubation, library clones were screened for EDO activity using freshly prepared 1% (vol/vol) catechol spray. Positive clones were identified by the formation of yellow 2-hydroxymuconic semialdehyde. Fosmids from two positive clones, SD3 and RW1 from sewage sludge and river water, respectively, were isolated using a FosmidMAX DNA purification kit (Epicentre) and sequenced by InterpretOmics India Pvt. Ltd. (Bengaluru, India) using an Illumina MiSeq platform. Reads obtained after sequencing were assembled using the CLC Genomics Workbench (Qiagen, Germany) using default parameters. Open reading frames (ORFs) were identified using the WEBMGA server (68) with the MetaGene program (69) and six-reading-frame technique. Blast2GO was also used to find prokaryotic genes (70). Functional annotation of proteins was done using CloudBlast on Blast2GO software. The genetic organizations of fosmids were visualized using DNAPlotter (71).

For evolutionary studies of the resulting protein sequences, the closest orthologs were identified using the blastp tool (NCBI), and the amino acid sequences were aligned using ClustalW (72). Phylogenetic trees were constructed using MEGA, version 7.0, software with bootstrap values calculated from 500 replicate runs (http://www.megasoftware.net/) (73). The evolutionary history was inferred using the neighbor-joining method (74). The evolutionary distances were calculated using the Poisson correction method (75) and are in the units of the number of amino acid substitutions per site.

### Recombinant plasmid construction.

The gene-specific primers for BphC-SD3 (forward, GCTCATATGATTCGTTCCATGGCATATCT; reverse, GCTACTCGAGTCAGTTTTCGATAATCATCGCCGG) and C23O-RW1, (forward, GCTCATATGAGCATCCCCTTCCGCT; reverse, GCTACTCGAGTCATGTCCGCGCCTCCG) were used to amplify genes using Phusion polymerase (Thermo Scientific). The PCR was run for 30 cycles with an annealing temperature of 60°C. The PCR product was digested using NdeI and XhoI (Thermo Scientific) and purified using a QIAquick Gel Extraction kit (Qiagen). Purified PCR product was ligated into a predigested (NdeI/XhoI) pET28a vector with an N-terminal 6×His tag sequence using T4 DNA ligase (Thermo Scientific), and the reaction mixture was incubated at 22°C for 1 h. The ligation product was transformed into Escherichia coli DH5α. The transformants were screened by colony PCR using T7 promoter and T7 terminator primers and DreamTaq polymerase (Thermo Scientific). Positive clones were further confirmed by DNA sequencing using an ABI capillary Genetic Analyzer 16.

### Protein overexpression and purification.

To purify recombinant proteins, E. coli BL21(DE3) cells harboring expression constructs were cultured in 5 ml of LB medium containing 35 μg/ml kanamycin at 37°C for 8 h, and then 2 ml was inoculated into 750 ml of LB medium with 35 μg/ml kanamycin. Cells were allowed to grow at 37°C until an optical density at 600 nm (OD_600_) of 0.6 was reached, and then cells were induced with 0.5 mM isopropyl-β-D-thiogalactopyranoside (IPTG) and reincubated at 37°C for 4 h. Cells were harvested by centrifugation at 5,000 × *g* for 15 min at 4°C, and the pellets were finally frozen at –80°C until use. For primary confirmation of dioxygenase activity, 50 μl of cells induced with 0.5 mM IPTG was spread on LB plates supplemented with 35 μg/ml kanamycin and incubated at 37°C overnight. The colonies were then sprayed using freshly prepared 1% (vol/vol) catechol, and dioxygenase activity was confirmed by the formation of yellow 2-hydroxymuconic semialdehyde (76).

For enzyme purification, the pellets of E. coli harboring the cloned gene were suspended in 20 mM Tris (pH 8)–250 mM NaCl buffer and lysed by ultrasonication at 4°C for 50 min. The lysate was then centrifuged at 12,000 × *g* for 40 min at 4°C, and the supernatant was used for further purification. The supernatant was loaded onto a Ni-NTA (1 ml) column preequilibrated with the above buffer. The enzyme was eluted with different concentrations of imidazole ranging from 20 mM to 500 mM. Elution fractions were analyzed by sodium dodecyl sulfate-polyacrylamide gel electrophoresis (SDS-PAGE) (77). The acrylamide concentrations for the stacking and separating gels were 5% and 15%, respectively. The gel was stained with Coomassie brilliant blue R250 (Bio-Rad). Protein concentrations were determined using the Bradford method (78). The protein was further purified using gel filtration chromatography with a Superdex S-200 10/300 GL increase column (GE Healthcare) preequilibrated with 20 mM Tris (pH 8)–250 mM NaCl buffer. The column was operated at a flow rate of 0.5 ml/min. Enzyme fractions were checked by SDS-PAGE, and, finally, the purified protein was concentrated to about 3 mg/ml using Amicon ultracentrifugal filters (Merck, Darmstadt, Germany). The oligomeric state of the protein was determined using an analytical Superdex S-200 10/300 GL increase column (GE Healthcare) which was calibrated with low-molecular-weight calibration standards (GE Healthcare).

### Biochemical characterization.

All enzyme activity measurements were performed in triplicates. The enzyme activity was determined by monitoring the formation of products at the enzyme’s respective wavelength (2,3-dihydroxybiphenyl at 434 nm; catechol at 375 nm; 3-methylcatechol at 388 nm; 4-methylcatechol at 382 nm; 4-chlorocatechol at 379 nm; pyrogallol at 375 nm, 1,2-dihydroxynaphthalene at 331 nm; 3-chlorocatechol at 290 nm) (17, 48). Measurements were carried out in 20 mM Tris-HCl buffer at 30°C and pH 8.0. Kinetic parameters of BphC-SD3 were determined at substrate ranges of 5 μM to 50 μM by using previously described extinction coefficients of reaction products (2,3-dihydroxybiphenyl, ε_434_ = 17,900 M^−1^ cm^−1^ [17]; catechol, ε_375_ = 14,700 M^−1^ cm^−1^ [79]; 3-methylcatechol, ε_388_ = 13,800 M^−1^ cm^−1^ [80]; 4-chlorocatechol, ε_379_ = 39,600 M^−1^ cm^−1^ [81]). Kinetic data were calculated from the initial velocities with the Michalis-Menten equation by nonlinear regression. One unit of enzyme activity was defined as the amount of enzyme that converted 1 μmol of substrate in 1 min under standard conditions. Optimum pH was determined by examining the activity under pH conditions ranging from pH 3 to pH 12 at an interval of 1.0 pH unit. Different buffers were used to provide the desired pH for the assay. Citrate buffer was used for pH 3 to 6, Tris buffer was used for pH 7 and 8, sodium bicarbonate buffer was used for pH 9 to 11, and potassium chloride sodium hydroxide buffer was used for pH 12. The effect of temperature on enzyme activity was examined by incubating the reaction mixture and enzyme individually at temperatures ranging from 5°C to 65°C for 30 min. Postincubation, components were mixed and examined for their activity. Halotolerance of enzymes was measured by increasing NaCl concentrations in the mixture for the reaction assay. The enzyme was preincubated with different concentrations of NaCl (0 to 4 M) for 30 min, and substrate was added postincubation to measure the enzyme activity. A one-way analysis of variance (ANOVA) was performed to compare the enzyme activities in the presence of different salt concentrations. As catechol dioxygenases are known to be active only in the presence of metal ions in their reaction centers, the relative activity in the presence of different metal ions was studied by replacing metal ions in the active site. The metal ions analyzed were Fe^2+^, Cs^+^, Cd^2+^, Co^2+^, Mn^2+^, Rh^3+^, Ca^2+^, Hg^2+^, Mg^2+^, Ni^2+^, Fe^3+^, Cu^2+^, and Zn^2+^. Purified protein (0.5 mg/ml) was first incubated with 5 mM EDTA overnight and dialyzed against Tris-NaCl buffer (pH 8) until no residual activity was observed. The resulting apoenzyme was then incubated for 15 min in the presence of different metal ions at a 1 mM final concentration, and enzyme activity was measured by adding substrate.

### AUC of BphC-SD3.

A Beckman-Coulter XL-A analytical ultracentrifuge equipped with a TiAn50 eight-hole rotor was used for analytical ultracentrifugation (AUC) experiments to determine the oligomeric state of BphC-SD3 in solution. The sedimentation velocity experiments were performed using two-channel epon centrepieces (12 mm) and quartz windows. Three different concentrations (8, 16 and 32 μM) of protein samples (in the same buffer as used in the SEC experiments) were run, and absorbance scans were recorded at 15°C at 280 nm at an interval of every 3 or 4 min with a speed of 40,000 rpm. SEDNTERP was used to calculate solvent density (ρ) and viscosity (η) from the chemical composition of proteins and buffers (82). A continuous size distribution model, *c*(*s*), was used to fit multiple scans at regular intervals with SEDFIT (29).

### Crystallization of BphC-SD3.

Commercial crystallization screens from Hampton Research, California, and Molecular Dimensions Limited, Suffolk, United Kingdom, were used to perform crystallization trials using a 0.5 mg/ml concentration of BphC-SD3 solubilized in 20 mM Tris (pH 8.0)–250 mM NaCl buffer. The crystallization trials were set up in two-drop 96-well high-throughput MRC plates using an NT8 automatic drop-setting robot (Formulatrix, Ltd., USA) by mixing 0.15 μl of protein and 0.15 μl of the precipitant solution and incubated at 20°C. Crystals appeared in the next 2 to 3 days.

### Data collection and processing of BphC-SD3.

The X-ray diffraction data for the BphC-SD3 crystal was collected using an in-house MAR345dtb image plate detector mounted on a Rigaku Micromax-007 HF rotating anode X-ray generator. The crystal was directly mounted on the beam and flash frozen in a cryo-stream at 100 K. The data were collected at a wavelength of 1.5418 Å. Each image was exposed for 10 min with 1° oscillation. The data were processed using iMOSFLM (83) and scaled using SCALA (84). The BphC-SD3 crystallized in tetragonal space group I422 with unit cell parameters of *a* = 98.64 Å, *b* = 98.64 Å, and *c* = 158.56 Å.

### Structure determination and refinement of BphC-SD3.

The structure of BhpC-SD3 was solved by the molecular replacement method using PHASER (85). The biphenyl-cleaving extradiol dioxygenase of *P. xenovorans* (PDB ID 1HAN, 40% sequence identity) was used as a search model (25). PHASER (85) with default parameters gave a single solution with one molecule of BphC-SD3 in the asymmetric unit. The model was refined using REFMAC5 (86), and iterative rounds of model building and restrained refinement were carried out using COOT (87) and REFMAC5, respectively. The final model has *R* and *R*_free_ values of 0.194 and 0.248, respectively. The model has excellent Ramachandran plot statistics, with 97% of residues in the favored region and 3% of residues in the allowed region.

### Electrochemical detection of the aromatic substrates.

Electrochemical experiments were performed using a PalmSens electrochemical workstation controlled by the software PS-Trace (PalmSens; Netherlands). Screen-printed electrodes (SPE) based on a standard three-electrode configuration (working, counter, and reference) with silver tracks were used. Cyclic voltammetry experiments were carried out in 50 mM phosphate-buffered saline (PBS) buffer, pH 8, at a scan rate of 100 mV/s. An enzyme concentration of 50 nM along with 500 μM substrate (catechol and 3-methylcatechol) was used for all experiments. Preliminary studies were performed for the detection of catecholic compounds. For the optimization of the substrate, catechol and 3-methylcatechol in the range of 200 μM to 700 μM were used to check the corresponding oxidation and reduction peaks.

### Data availability.

The sequence information of fosmid clones BphC and RW1 have been submitted to the NCBI Sequence Read Archive (SRA) under accession numbers SRR8739405 and SRR8755636, respectively. The gene sequences corresponding to BphC-SD3 and C23O-RW1 have been submitted to GenBank under accession numbers KX965751 and KX965752, respectively. Structural data are available in the RCSB PDB database under the PDB ID 6L3W.
