# Supplementary figures and images for: Structure Elucidation and Biochemical Characterization of Environmentally Relevant Novel Extradiol Dioxygenases Discovered by a Functional Metagenomics Approach
Source: mSystems. 2019 Nov 26;4(6):e00316-19. doi: 10.1128/mSystems.00316-19 (PMC6880040; doi:10.1128/mSystems.00316-19)

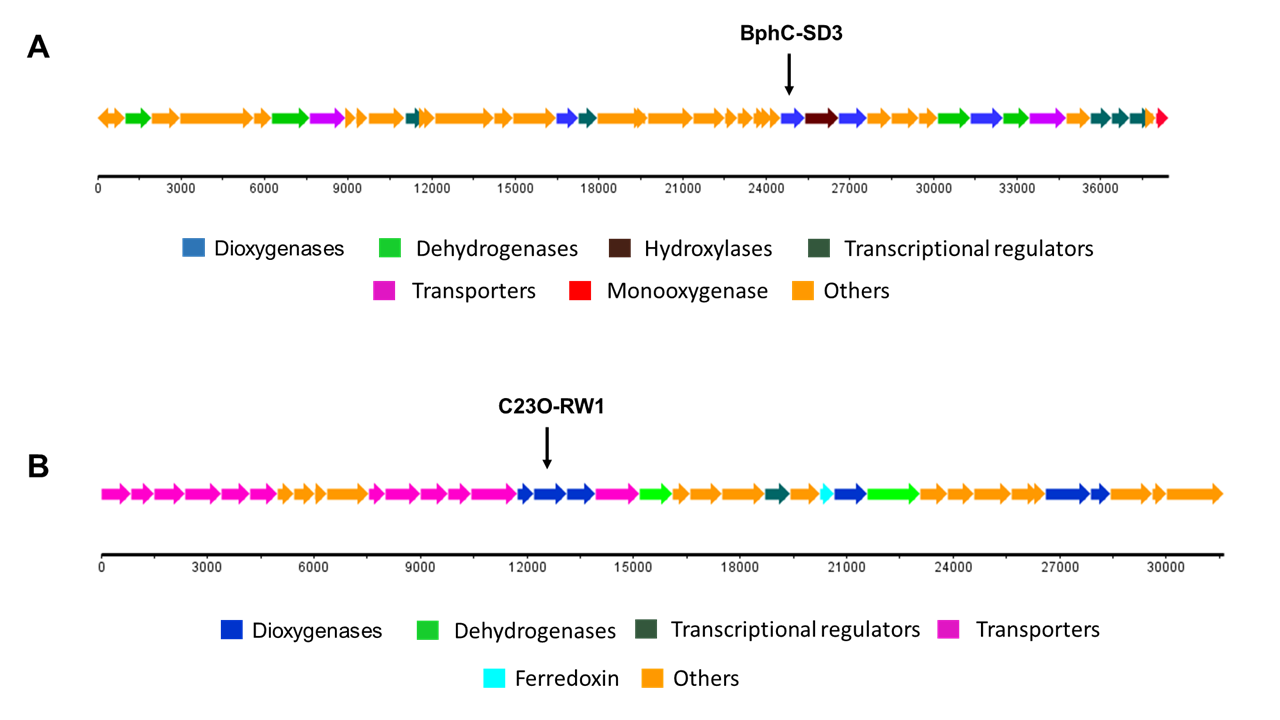

Supplement: FIG S1 [file mSystems.00316-19-sf001.tif]

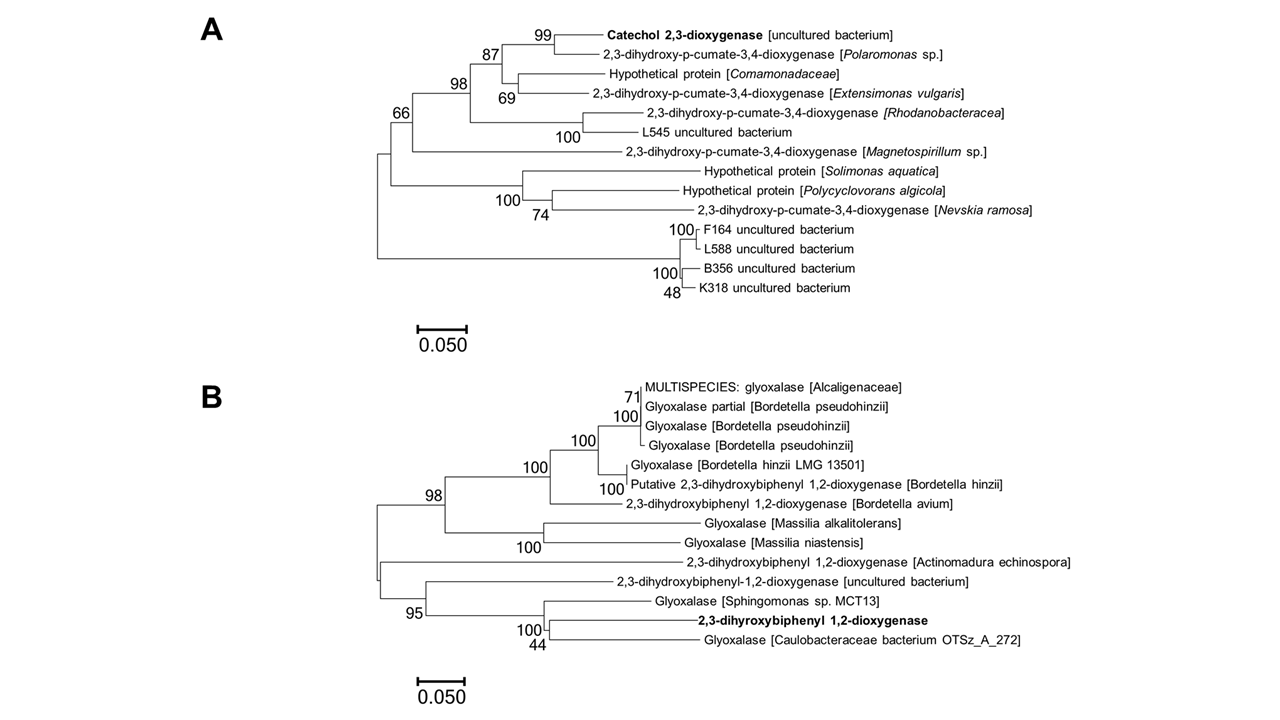

Supplement: FIG S2 [file mSystems.00316-19-sf002.tif]

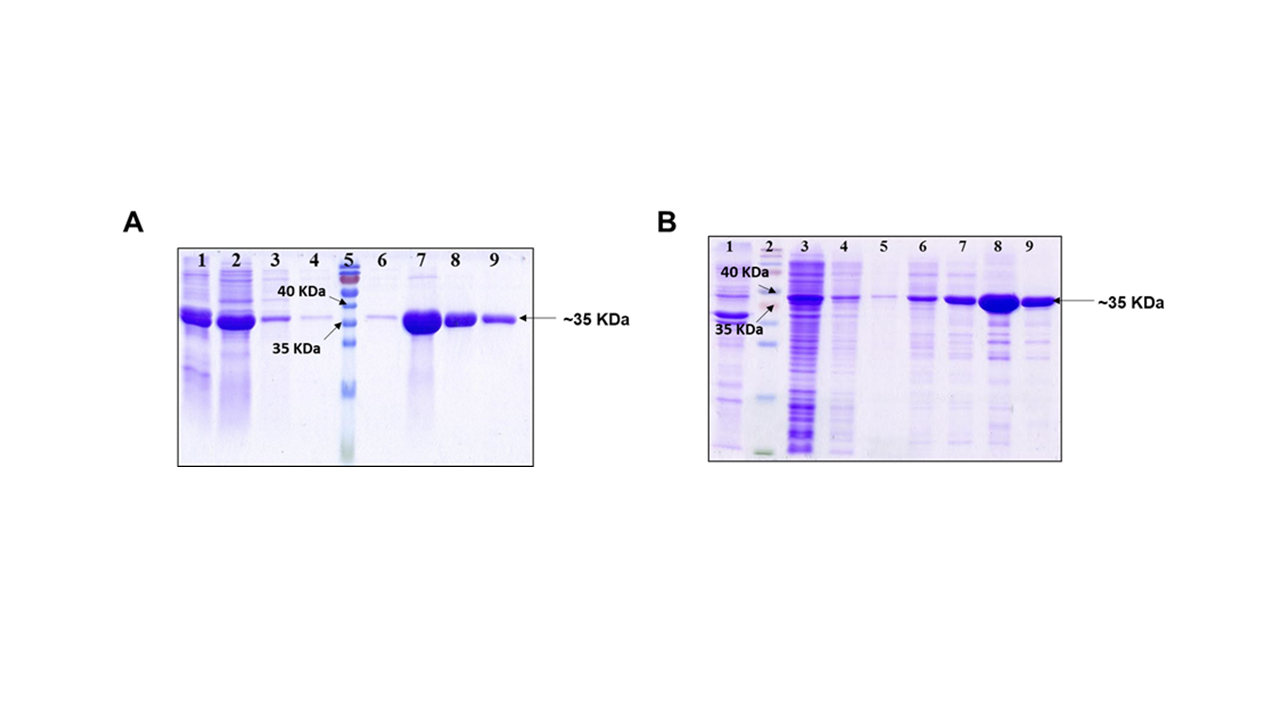

Supplement: FIG S3 [file mSystems.00316-19-sf003.tif]

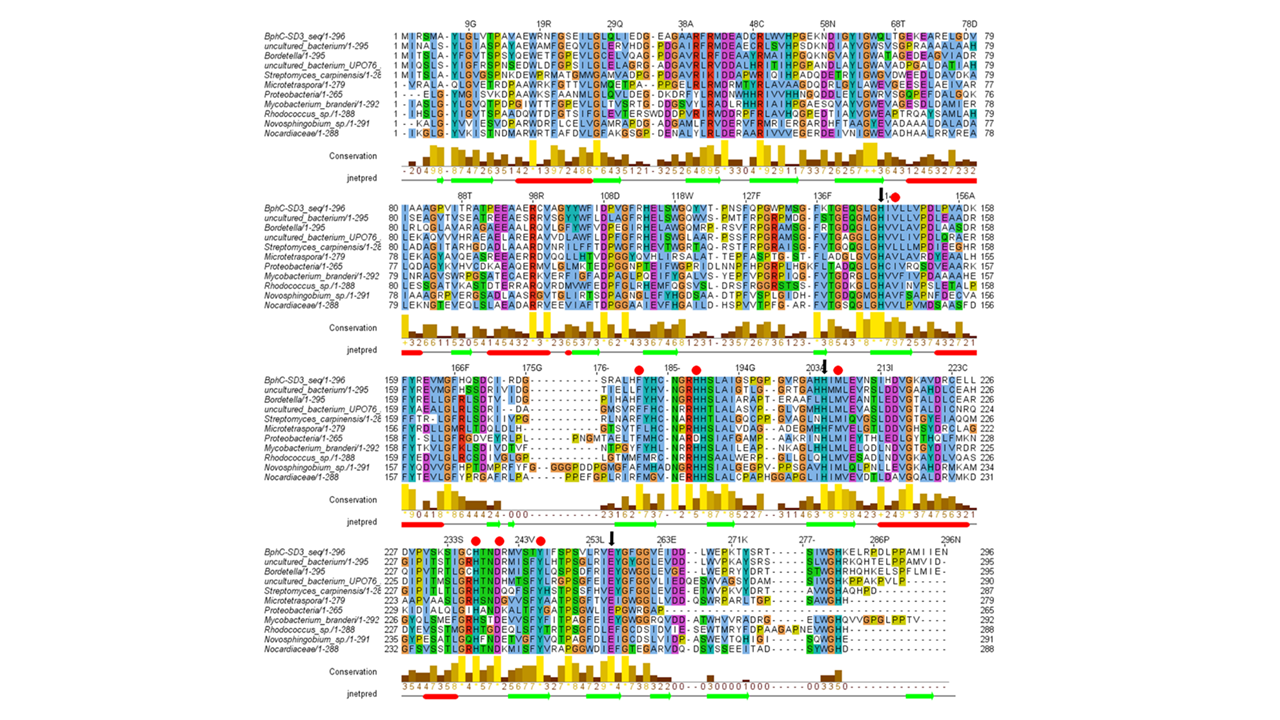

Supplement: FIG S4 [file mSystems.00316-19-sf004.tif]

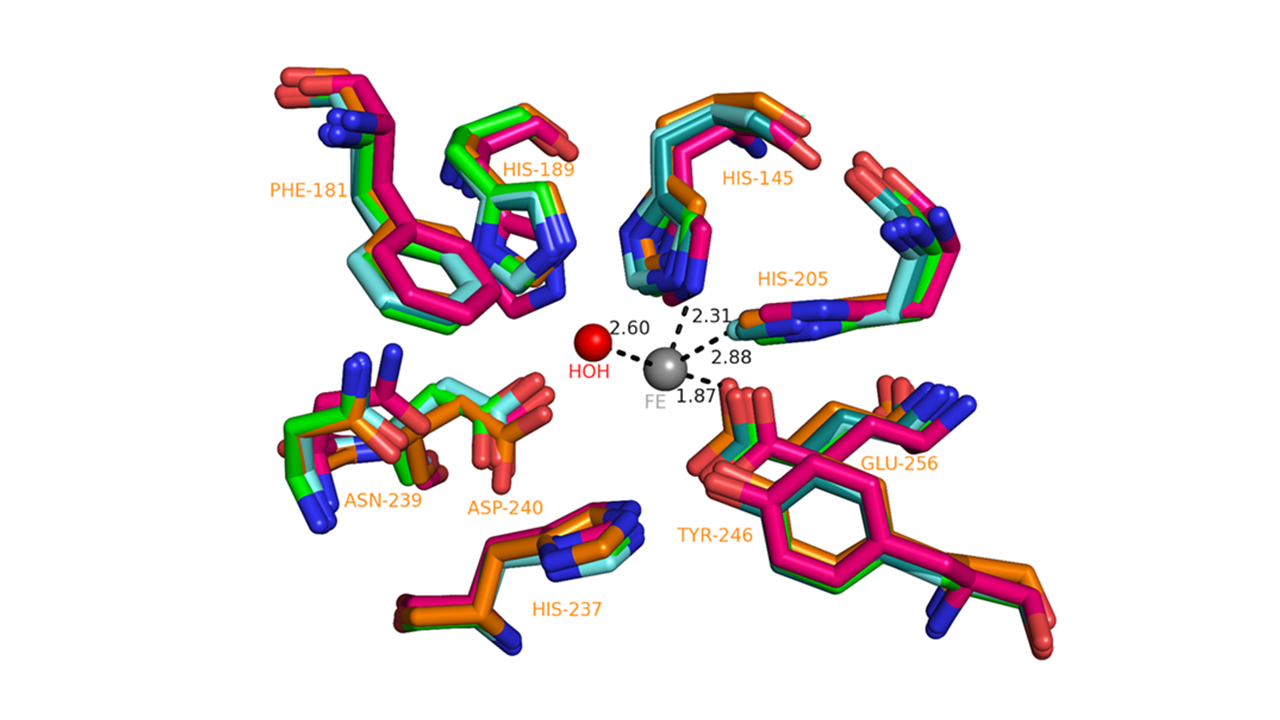

Supplement: FIG S5 [file mSystems.00316-19-sf005.tif]

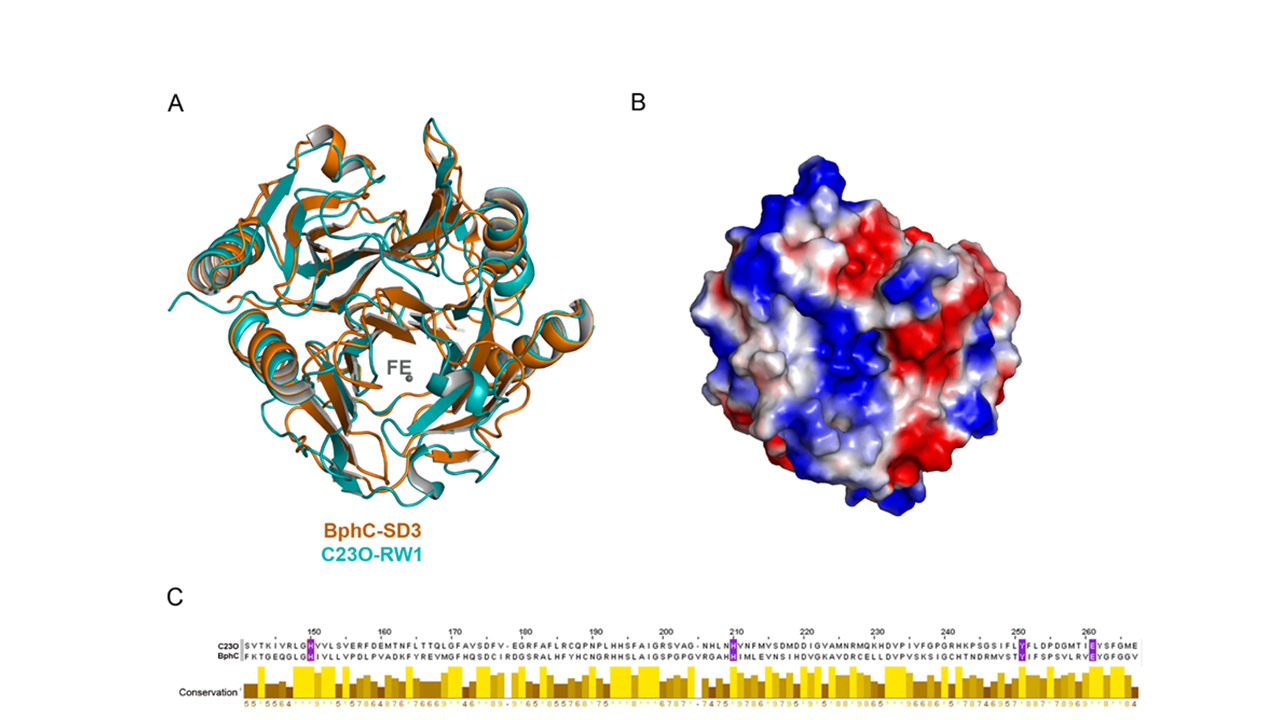

Supplement: FIG S6 [file mSystems.00316-19-sf006.tif]

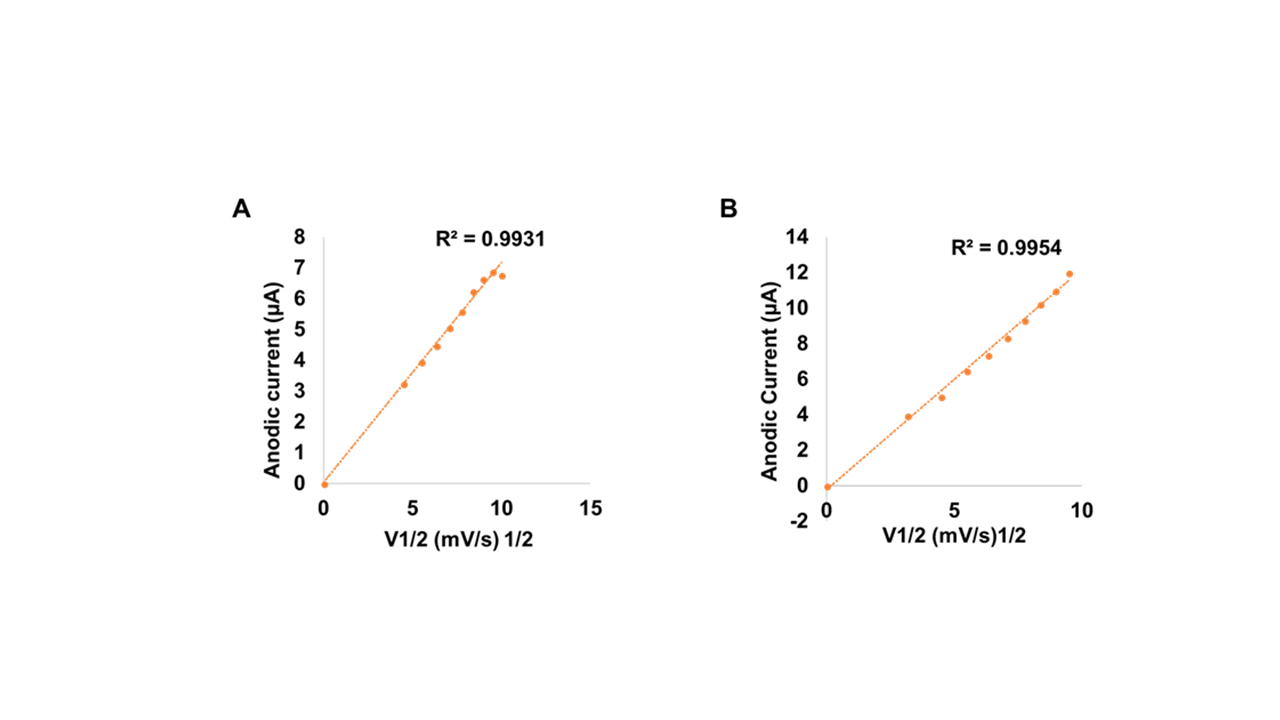

Supplement: FIG S7 [file mSystems.00316-19-sf007.tif]
