# Supplementary material for: Structure Elucidation and Biochemical Characterization of Environmentally Relevant Novel Extradiol Dioxygenases Discovered by a Functional Metagenomics Approach
Source: mSystems. 2019 Nov 26;4(6):e00316-19. doi: 10.1128/mSystems.00316-19 (PMC6880040; doi:10.1128/mSystems.00316-19)
Supplement: TABLE S1 [file mSystems.00316-19-st001.docx]

**Table S1**

| **S.NO.** | **SEQUENCE DESCRIPTION** | **LENGTH (bp)** |
| --- | --- | --- |
| 1 | Thiol-disulfide oxidoreductase [*Gluconobacter oxydans*] | 207 |
| 2 | 2-keto-4-pentenoate hydratase [*Sphingopyxis macrogoltabida*] | 783 |
| 3 | Acetaldehyde dehydrogenase (acetylating) | 942 |
| 4 | 4-hydroxy-2-oxovalerate aldolase | 1032 |
| 5 | Phosphate dikinase | 2661 |
| 6 | Asp Glu hydantoin racemase | 645 |
| 7 | Glutamate dehydrogenase | 1362 |
| 8 | Long-chain fatty acid transporter | 1263 |
| 9 | Transporter component [*Serratia marcescens*] | 408 |
| 10 | Membrane [*Methylobacterium extorquens*] | 417 |
| 11 | Ubiquinol-cytochrome C reductase | 1323 |
| 12 | Family transcriptional regulator | 735 |
| 13 | ---NA--- | 144 |
| 14 | Thiol-disulfide oxidoreductase | 198 |
| 15 | Receptor | 2130 |
| 16 | Thiaminase II | 663 |
| 17 | Leucyl aminopeptidase | 1569 |
| 18 | Taurine dioxygenase | 789 |
| 19 | Crp Fnr family transcriptional regulator [*Sphingopyxis MC1*] | 657 |
| 20 | DEAD DEAH box helicase | 1722 |
| 21 | ---NA--- | 114 |
| 22 | MULTISPECIES: sulfatase [*Sphingopyxis*] | 1629 |
| 23 | Transferase [*Bordetella pseudohinzii*] | 1140 |
| 24 | Uncharacterised [*Achromobacter*] | 432 |
| 25 | ---NA--- | 585 |
| 26 | Membrane [*Bordetella avium*] | 477 |
| 27 | Membrane [*Bordetella pseudohinzii*] | 192 |
| 28 | Hypothetical protein [*Sphingomonas* sp. MCT13] | 297 |
| 29 | 2,3-dihydroxybiphenyl 1,2-dioxygenase | 864 |
| 30 | Hydroxylase [*Bordetella pseudohinzii*] | 1218 |
| 31 | Gentisate 1,2-dioxygenase [*Achromobacter*] | 1026 |
| 32 | 2-hydroxyhepta-2,4-diene-1,7-dioate isomerase | 888 |
| 33 | 4,5-dihydroxyphthalate decarboxylase | 978 |
| 34 | Membrane [*Bordetella avium*] | 672 |
| 35 | Acyl- dehydrogenase | 1173 |
| 36 | Iron-sulfur containing oxygenase | 1176 |
| 37 | Short-chain dehydrogenase [*Bordetella pseudohinzii*] | 933 |
| 38 | MFS transporter | 1347 |
| 39 | Alpha beta hydrolase [*Burkholderia pseudomultivorans*] | 843 |
| 40 | Transcriptional regulator [*Burkholderia pseudomultivorans*] | 756 |
| 41 | Family transcriptional regulator | 651 |
| 42 | Family transcriptional regulator | 819 |
| 43 | ---NA--- | 144 |
| 44 | 4-hydroxyphenylacetate 3-monooxygenase [*Burkholderia seminalis*] | 453 |
