# Supplementary material for: Structure Elucidation and Biochemical Characterization of Environmentally Relevant Novel Extradiol Dioxygenases Discovered by a Functional Metagenomics Approach
Source: mSystems. 2019 Nov 26;4(6):e00316-19. doi: 10.1128/mSystems.00316-19 (PMC6880040; doi:10.1128/mSystems.00316-19)
Supplement: TABLE S2 [file mSystems.00316-19-st002.docx]

**Table S2**

| **S.NO.** | **SEQUENCE DESCRIPTION** | **LENGTH (bp)** |
| --- | --- | --- |
| 1 | Branched-chain amino acid ABC transporter permease [*Bordetella bronchiseptica*] | 864 |
| 2 | ABC transporter substrate-binding | 648 |
| 3 | MULTISPECIES: branched-chain amino acid ABC transporter permease [*Janthinobacterium*] | 873 |
| 4 | Branched-chain amino acid ABC transporter permease | 999 |
| 5 | ABC transporter ATP-binding | 807 |
| 6 | ABC transporter ATP-binding | 774 |
| 7 | Phenylacetic acid degradation | 465 |
| 8 | Hemerythrin | 585 |
| 9 | ---NA--- | 363 |
| 10 | ABC transporter substrate-binding | 1161 |
| 11 | ABC transporter substrate-binding | 459 |
| 12 | Branched-chain amino acid ABC transporter permease | 1017 |
| 1314 | ABC transporter ATP-binding [*Bordetella bronchiseptica*] | 762 |
| 15 | ABC transporter ATP-binding | 666 |
| 16 | p-cumate dioxygenase [*Variovorax paradoxus*] | 1275 |
| 17 | Aromatic-ring-hydroxylating dioxygenase | 492 |
| 18 | Catechol 2,3-dioxygenase | 912 |
| 19 | 2,3-dihydroxy-2,3-dihydro-p-cumate dehydrogenase | 813 |
| 20 | MFS transporter [*Serratia liquefaciens*] | 1239 |
| 21 | Acetaldehyde dehydrogenase (acetylating) | 915 |
| 22 | Ribosomal- -alanine N-acetyltransferase | 501 |
| 23 | Uracil-DNA glycosylase | 903 |
| 24 | A Chain Crystal Structure of Ferredoxin Bpha4 (Oxidized Form) | 1227 |
| 25 | transcriptional regulator | 708 |
| 26 | 2-hydroxy-6-oxo-2,4-heptadienoate hydrolase | 840 |
| 27 | Ferredoxin [*Variovorax paradoxus*] | 384 |
| 28 | Chlorocatechol 2,3-dioxygenase | 945 |
| 29 | 2-hydroxymuconic semialdehyde dehydrogenase | 1482 |
| 30 | 2-oxopent-4-enoate hydratase [*Variovorax paradoxus*] | 783 |
| 31 | tRNA (adenosine(37)-N6)-threonylcarbamoyltransferase complex dimerization subunit type 1 | 729 |
| 32 | 4-hydroxy-2-oxovalerate aldolase [*Burkholderia territorii*] | 1050 |
| 33 | 4-oxalocrotonate decarboxylase | 789 |
| 34 | 4-oxalocrotonate tautomerase | 171 |
| 35 | Ring-hydroxylating oxygenase subunit alpha | 1293 |
| 36 | Ibuprofen- dioxygenase small subunit | 537 |
| 37 | Sterol carrier | 1194 |
| 38 | Nucleic-acid-binding containing a Zn-ribbon [*Mycobacterium senegalense*] | 408 |
| 39 | AMP-dependent synthetase and ligase | 1587 |
