# Supplementary material for: Structure Elucidation and Biochemical Characterization of Environmentally Relevant Novel Extradiol Dioxygenases Discovered by a Functional Metagenomics Approach
Source: mSystems. 2019 Nov 26;4(6):e00316-19. doi: 10.1128/mSystems.00316-19 (PMC6880040; doi:10.1128/mSystems.00316-19)
Supplement: TABLE S3 [file mSystems.00316-19-st003.docx]

**Table S3 (a)**

| **Coordination distances (Å)** | |
| --- | --- |
| Fe-NE2 (His145) | 2.40 |
| Fe-NE2 (His205) | 2.33 |
| Fe-OE1 (Glu256) | 2.27 |
| Fe-Wat (HOH503) | 2.26 |
| **Bond angles (degree)** | |
| NE2 (His145)-Fe-NE2 (His205) | 107.93 |
| NE2 (His205)-Fe-OE1 (Glu256) | 88.72 |
| OE1 (Glu256)-Fe-NE2 (His145) | 93.93 |
| Wat (HOH503)-Fe-NE2 (His145) | 119.49 |
| Wat (HOH503)-Fe-NE2 (His205) | 108.12 |
| Wat (HOH503)-Fe-OE1 (Glu256) | 133.40 |

**Table S3 (b)**

| **Enzyme** | **Metal** | **Binding residues** | **PDB ID** |
| --- | --- | --- | --- |
| 2,3-dihydroxybiphenyl 1,2-dioxygenase from *P. cepacia* LB400 | Fe(II) | His-146, His-210 and Glu-260 | 1KND ([1](#_ENREF_1)) |
| 2,3-dihydroxybiphenyl dioxygenase (BphC) from *Pseudomonas sp.* strain KKS102 | Fe(III) | His-145, His-209 and Glu-260 | 1DHY ([2](#_ENREF_2)) |
| 2,3-dihydroxybiphenyl dioxygenase (BphC) from *Pseudomonas* sp. strain KKS102 | Fe(II) | His-145, His-209 and Glu-260 | 1KW9 ([3](#_ENREF_3)) |
| Catechol 2,3-dioxygenase (metapyrocatechase, MPC) from *Pseudomonas putida* mt-2 | Fe(II) | His-153, His-214 and Glu-265 | 1MPY ([4](#_ENREF_4)) |
| 2,3-dihydroxybiphenyl dioxygenase (BphC_JF8) from *Bacillus* sp. JF8 | Mn(II) | His-155, His-214 and Glu-266 | 3D-Model ([5](#_ENREF_5)) |

**References**

1. Vaillancourt FH, Han S, Fortin PD, Bolin JT, Eltis LD. 1998. Molecular basis for the stabilization and inhibition of 2, 3-dihydroxybiphenyl 1,2-dioxygenase by t-butanol. J Biol Chem 273:34887-95.

2. Senda T, Sugiyama K, Narita H, Yamamoto T, Kimbara K, Fukuda M, Sato M, Yano K, Mitsui Y. 1996. Three-dimensional structures of free form and two substrate complexes of an extradiol ring-cleavage type dioxygenase, the BphC enzyme from *Pseudomonas* sp. strain KKS102. J Mol Biol 255:735-52.

3. Sato N, Uragami Y, Nishizaki T, Takahashi Y, Sazaki G, Sugimoto K, Nonaka T, Masai E, Fukuda M, Senda T. 2002. Crystal structures of the reaction intermediate and its homologue of an extradiol-cleaving catecholic dioxygenase. J Mol Biol 321:621-36.

4. Kita A, Kita S, Fujisawa I, Inaka K, Ishida T, Horiike K, Nozaki M, Miki K. 1999. An archetypical extradiol-cleaving catecholic dioxygenase: the crystal structure of catechol 2,3-dioxygenase (metapyrocatechase) from *Pseudomonas putida* mt-2. Structure 7:25-34.

5. Hatta T, Mukerjee-Dhar G, Damborsky J, Kiyohara H, Kimbara K. 2003. Characterization of a novel thermostable Mn(II)-dependent 2,3-dihydroxybiphenyl 1,2-dioxygenase from a polychlorinated biphenyl- and naphthalene-degrading *Bacillus* sp. JF8. J Biol Chem 278:21483-92.
